# Supplementary material for: Proteogenomic landscape of squamous cell lung cancer
Source: Nat Commun. 2019 Aug 8;10:3578. doi: 10.1038/s41467-019-11452-x (PMC6687710; doi:10.1038/s41467-019-11452-x)
Supplement: Supplementary file 1 — Supplementary Information [file 41467_2019_11452_MOESM1_ESM.pdf]

Supplementary Information

Stewart *et al.*

Supplementary Figure 1

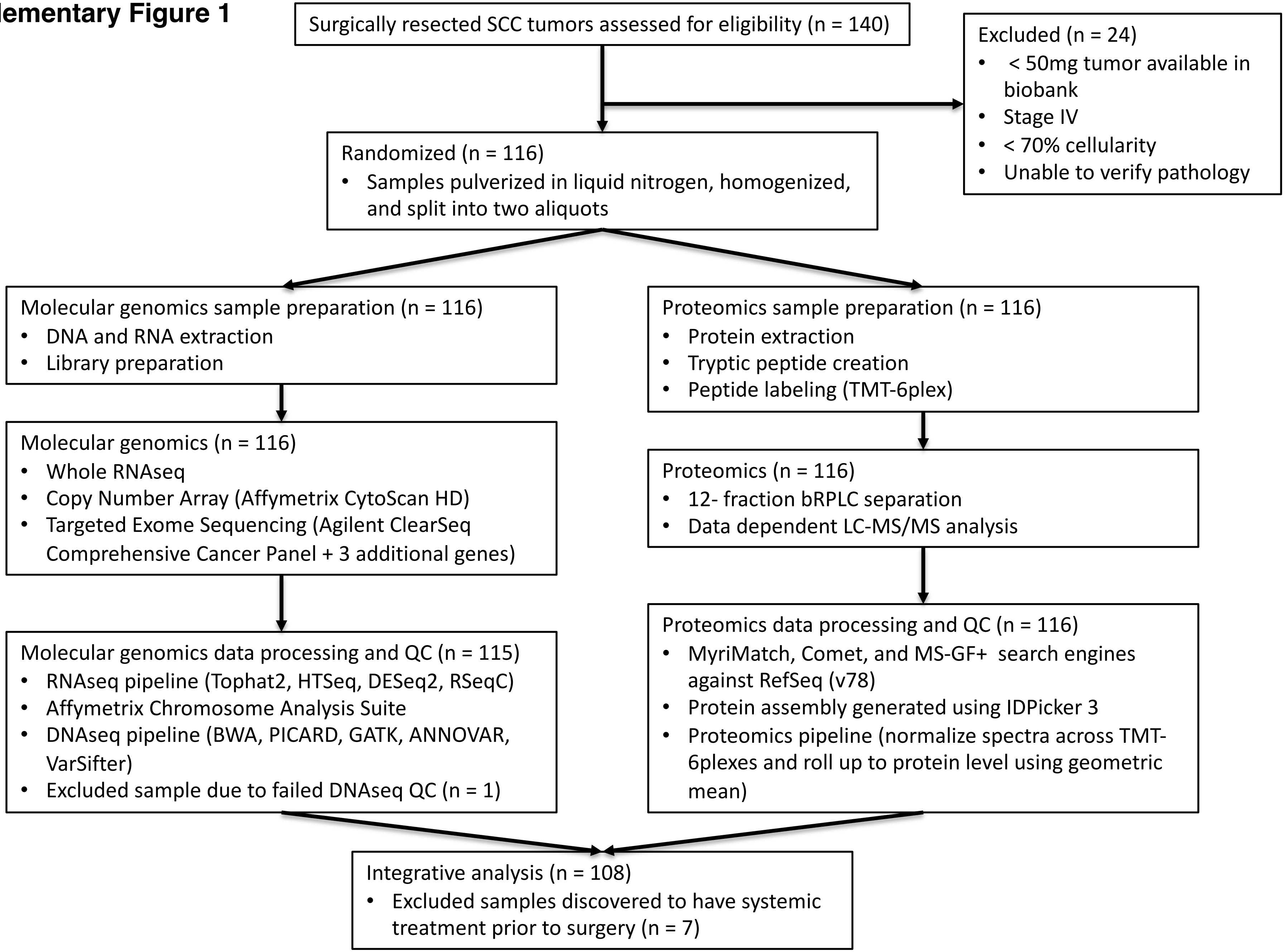

Supplementary Figure 1 – CONSORT flow diagram for sample exclusion and processing.

Supplementary Figure 2

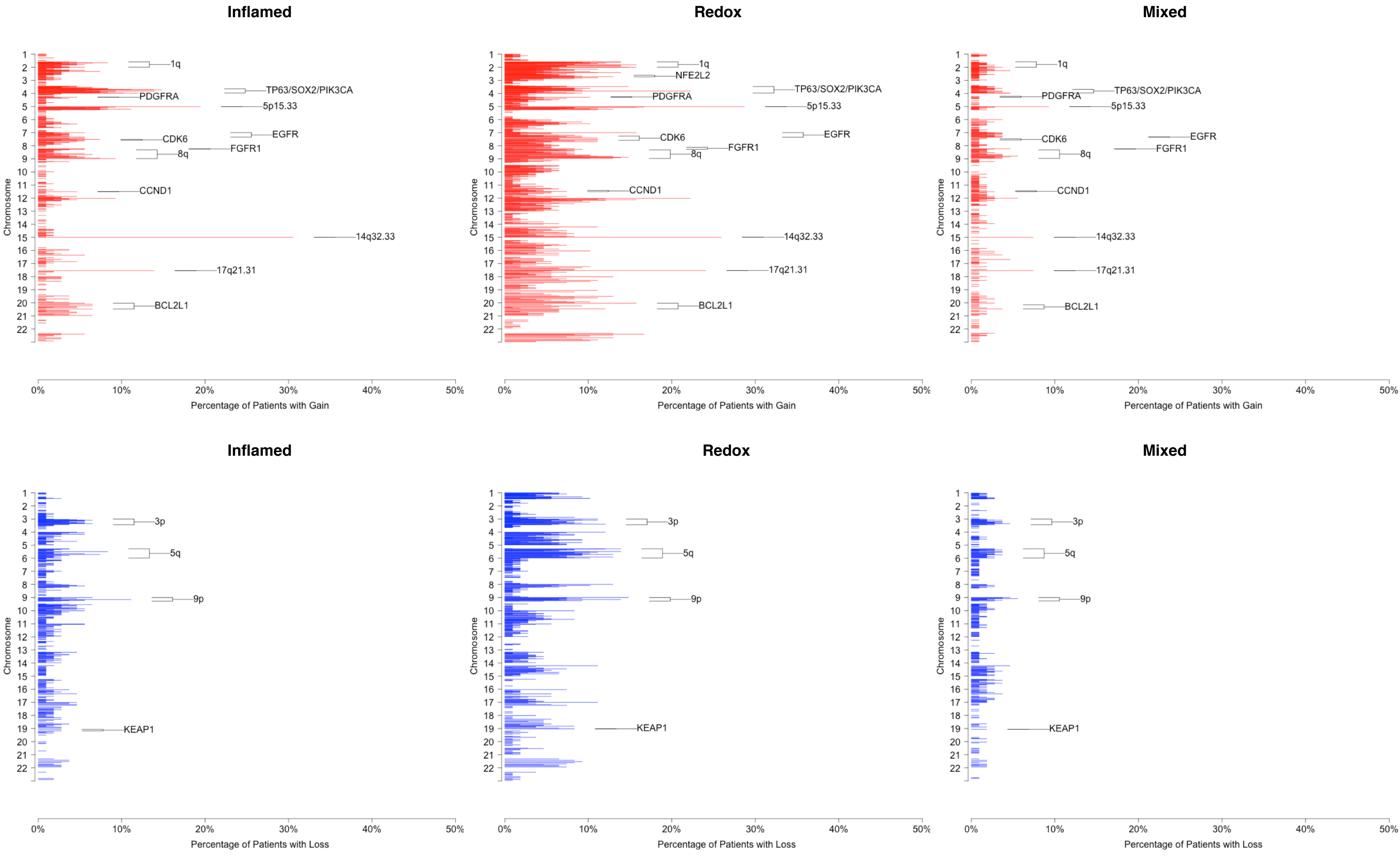

Supplementary Figure 2 – Copy number alterations by proteomic subtype. Chromosomes are arranged on the Y axis with the percent of patients with gains or losses of a particular region arranged along the X axis. Regions and genes are shown according to their relative position on the chromosome according to their genomic coordinates from hg19. For a given chromosome, the P arm is represented as the top portion of the Y axis and the Q arm is the bottom portion.

Supplementary Figure 3

A)

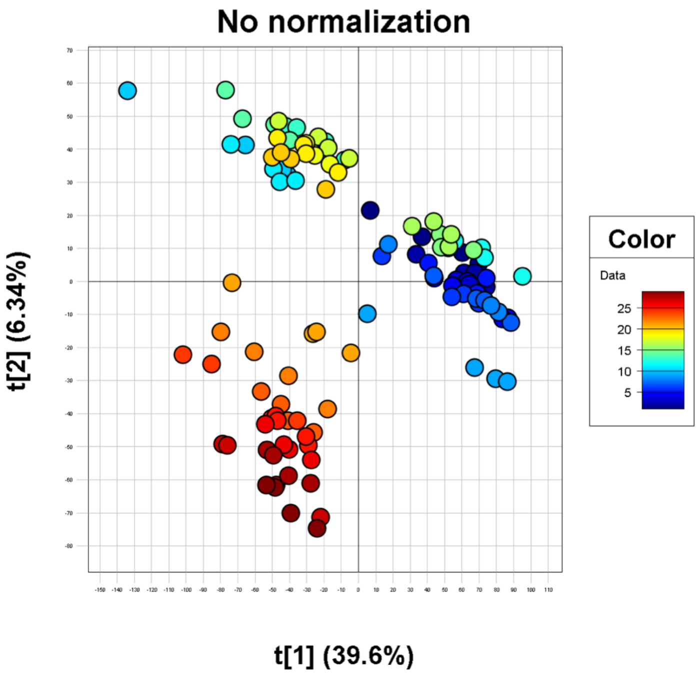

B)

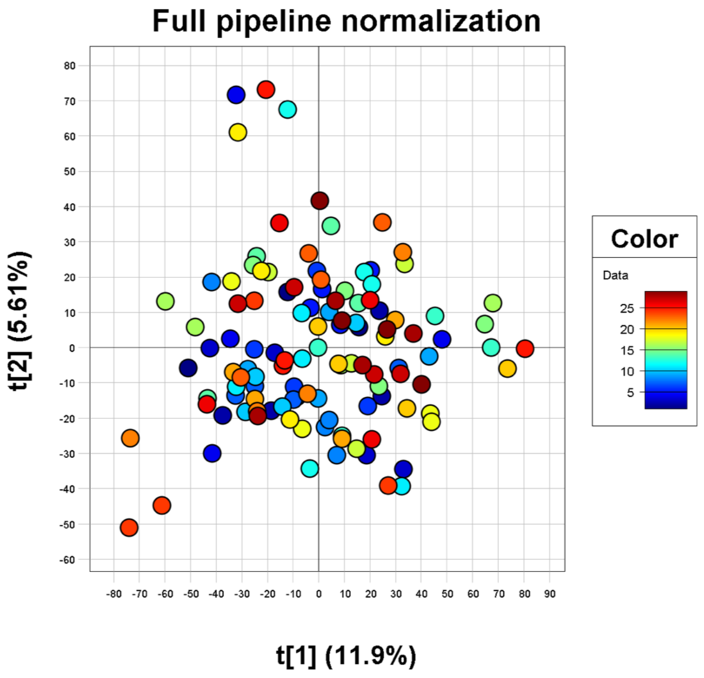

C)

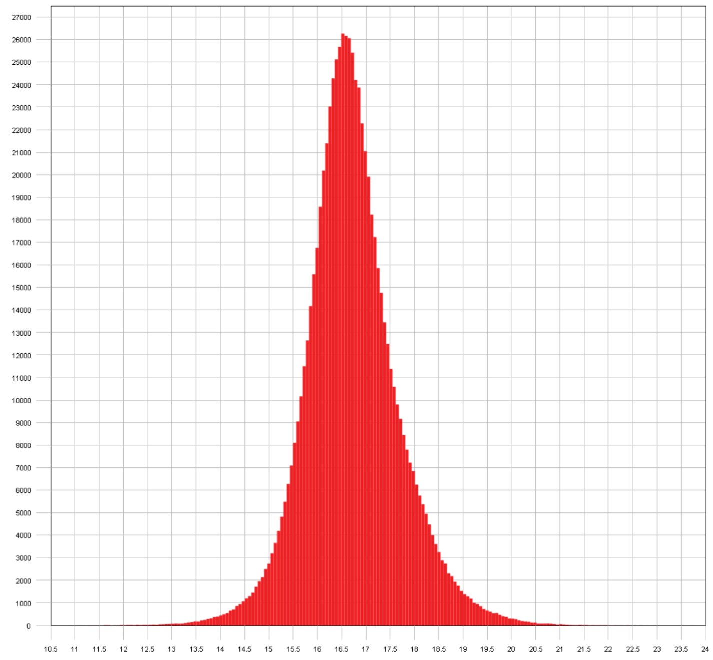

Supplementary Figure 3 – Effects of normalization on sample signal. Principal Component Analysis (PCA), using the NIPALS algorithm for treating missing data, was applied to the subset of tumor samples at various levels of post-processing. A) Peptides summarized at the protein-level, no normalization. Three major batches are observed (early, middle, late), with the early batch significantly brighter than the later batches. Within-plex tumor samples cluster with each other and separate from other 6-plexes within the same batch. B) Processing the samples with the full normalization pipeline removes both batch effect and between-plex differences. C)**.** Histogram of normalized $\log_2$ abundances. The global abundance distribution of all tumor samples exhibits a roughly log-normal distribution with a slight positive skewness. This positive skewness could be due to depletion of observed low-end abundances, which are more likely to be below the limit of detection within any given 6-plex.

Supplementary Figure 4

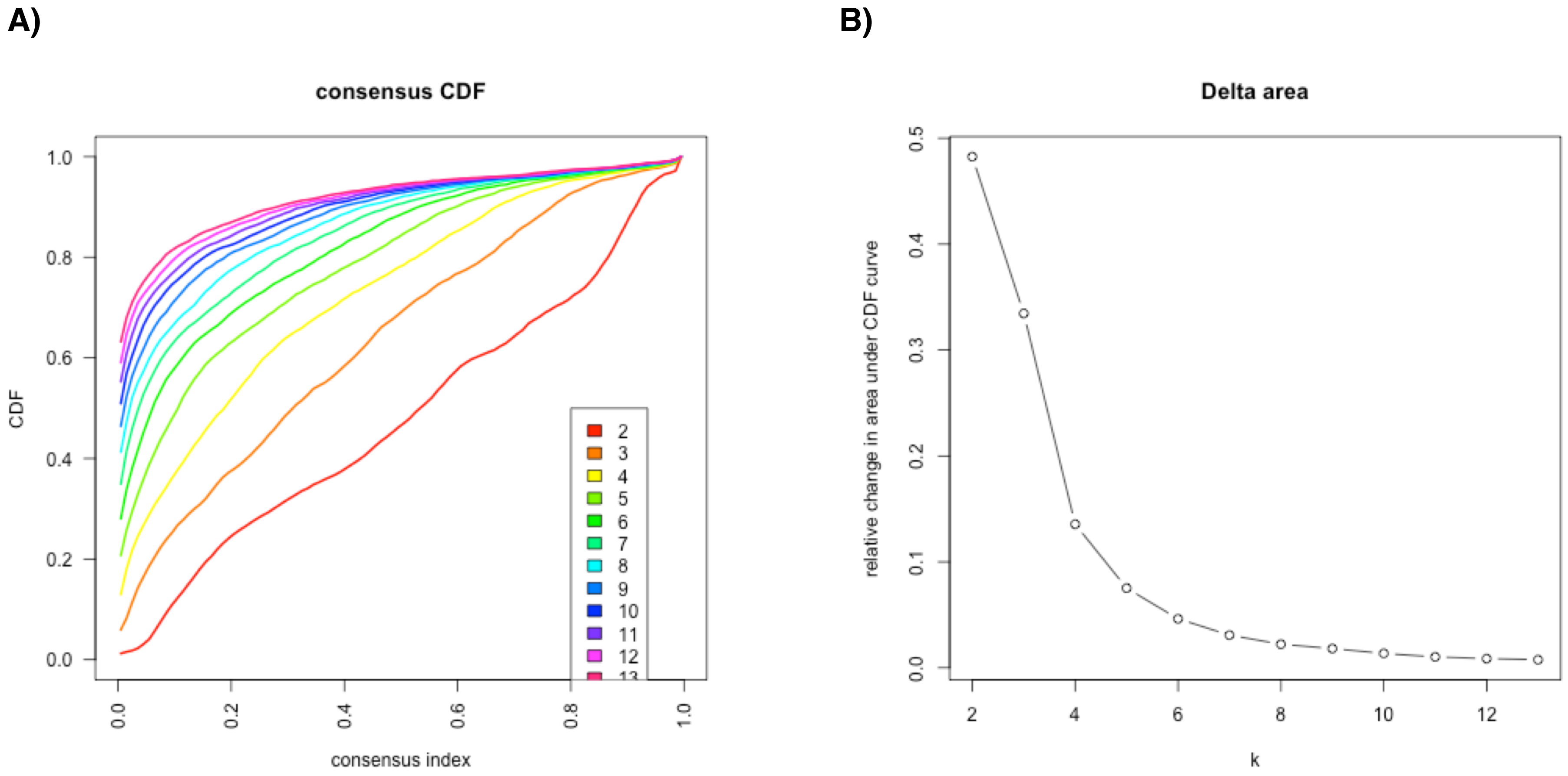

Supplementary Figure 4 – Proteomics consensus clustering diagnostic plots. A) Change in the cumulative distribution function (CDF) as the number of consensus cluster groups (k) is increased. B) The change in the area under each curve from A showing convergence at k = 5.

Supplementary Figure 5

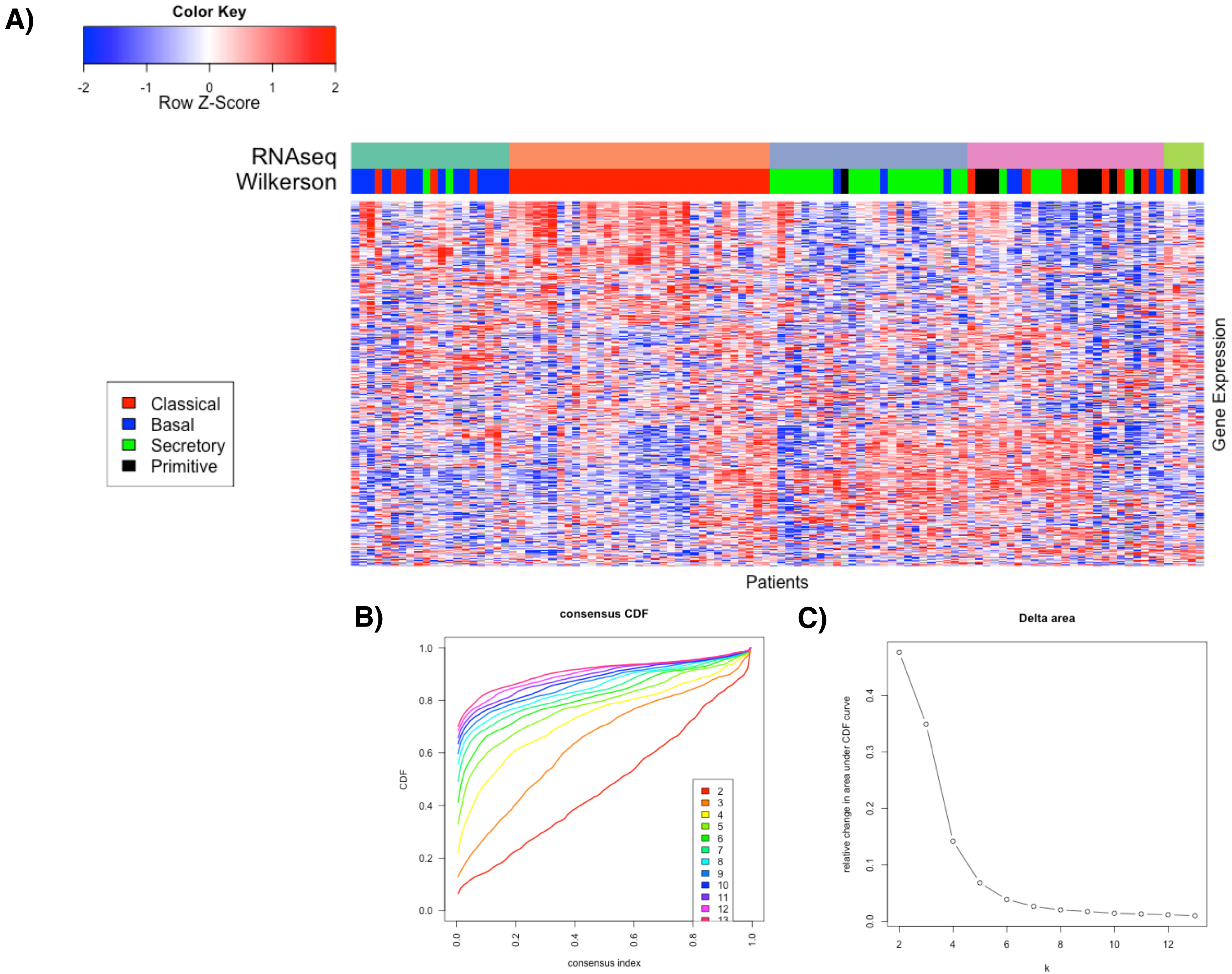

Supplementary Figure 5 – Consensus clustering of RNAseq expression. A) 108 patient tumors are displayed as columns, and the 1,000 most variable genes by absolute median deviation are displayed as rows. There is partial concordance with the Wilkerson *et al.* mRNA-based classifiers of these same samples, but the primitive group is not recapitulated. B) Change in CDF as the number of consensus cluster groups (k) is increased. C) The change in the area under each curve from B showing convergence at k = 5.

Supplementary Figure 6

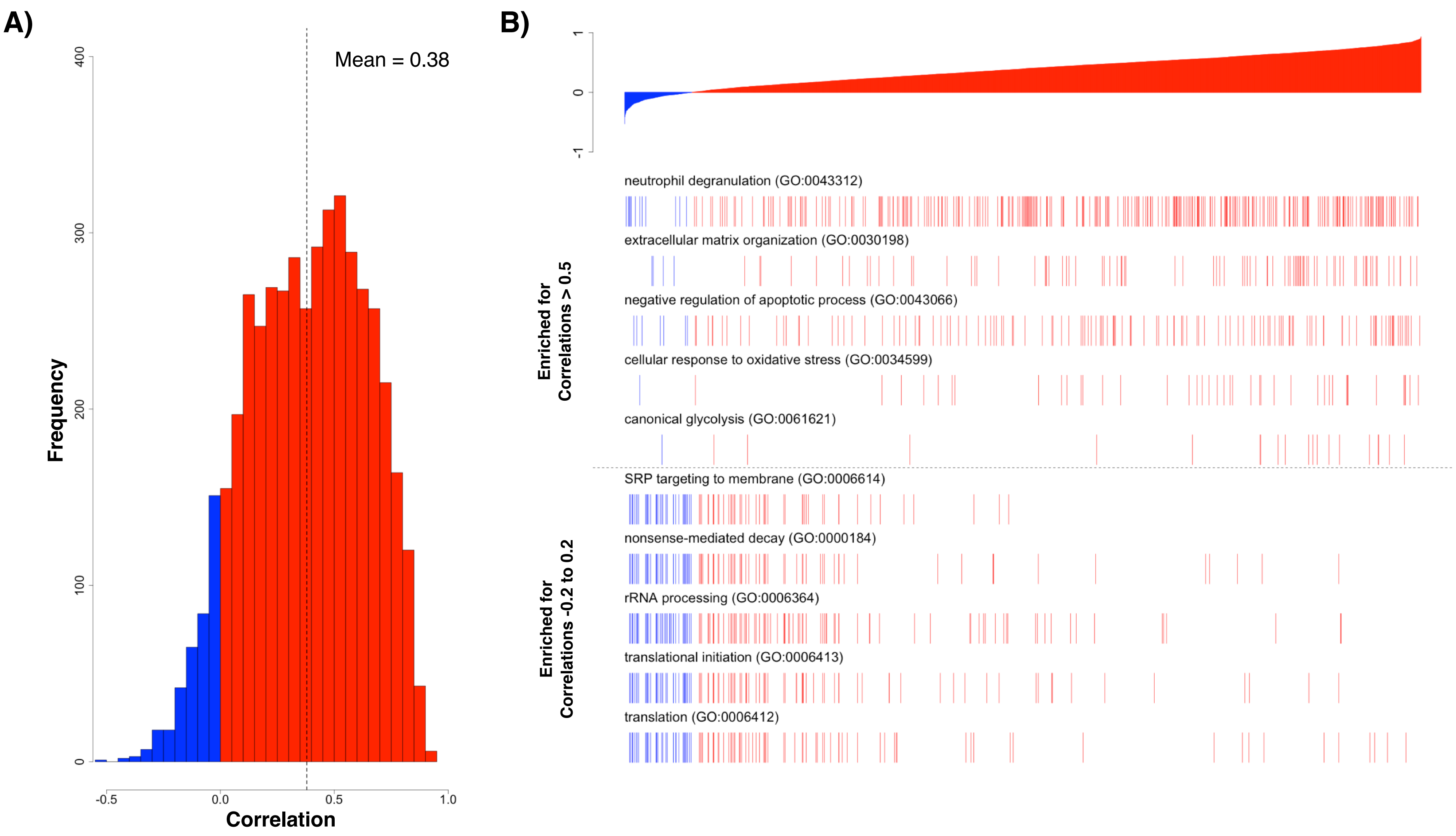

Supplementary Figure 6 – mRNA and protein correlations. A) Histogram showing 4,625 genes and correlated with matched protein expression. The mean transcript-protein pair correlation, 0.38, is consistent with other proteogenomic studies. B) Waterfall plot of the same 4,625 transcript-protein pairs (top). Highly correlated proteins are enriched for neutrophil and redox pathways (middle), while poorly correlated pathways are enriched for translational machinery and nonsense-mediated decay (bottom).

Supplementary Figure 7

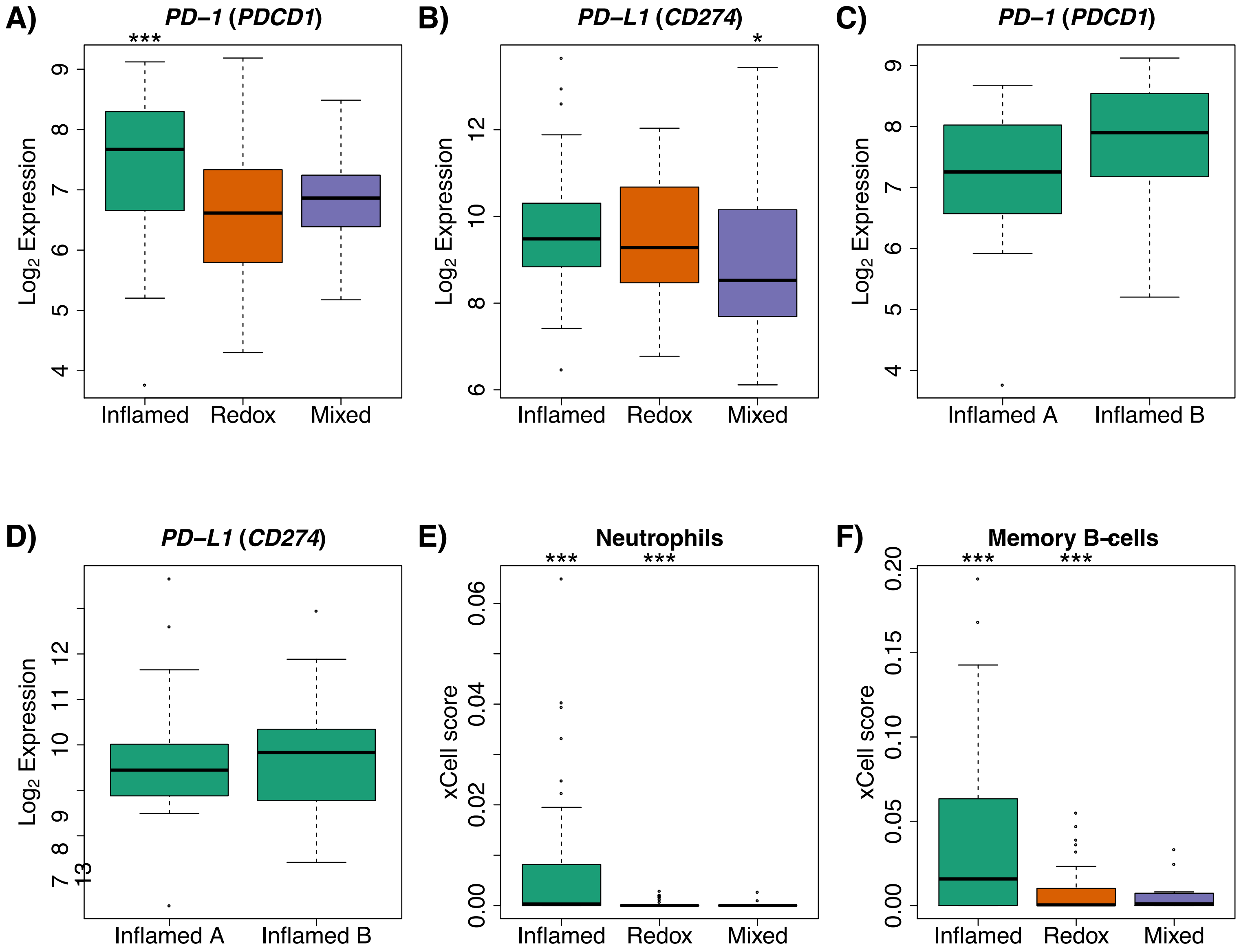

Supplementary Figure 7 – PD-1 expression, PD-L1 expression, and xCell scores. A) *PD-1* was significantly elevated in Inflamed at the RNA level (Wilcoxon P = 1.74E-04). B) *PD-L1* was significantly lower in Mixed at the RNA level (Wilcoxon P = 0.05). C) and D) There was no difference in *PD-1* or *PD-L1* levels between Inflamed A and Inflamed B. E) xCell neutrophil scores were significantly higher in Inflamed (Wilcoxon P = 2.083E-05) compared to the rest of the cohort. F) xCell memory B-cell scores were significantly higher in Inflamed (Wilcoxon P = 2.53E-04) compared to the rest of the cohort.

Supplementary Figure 8

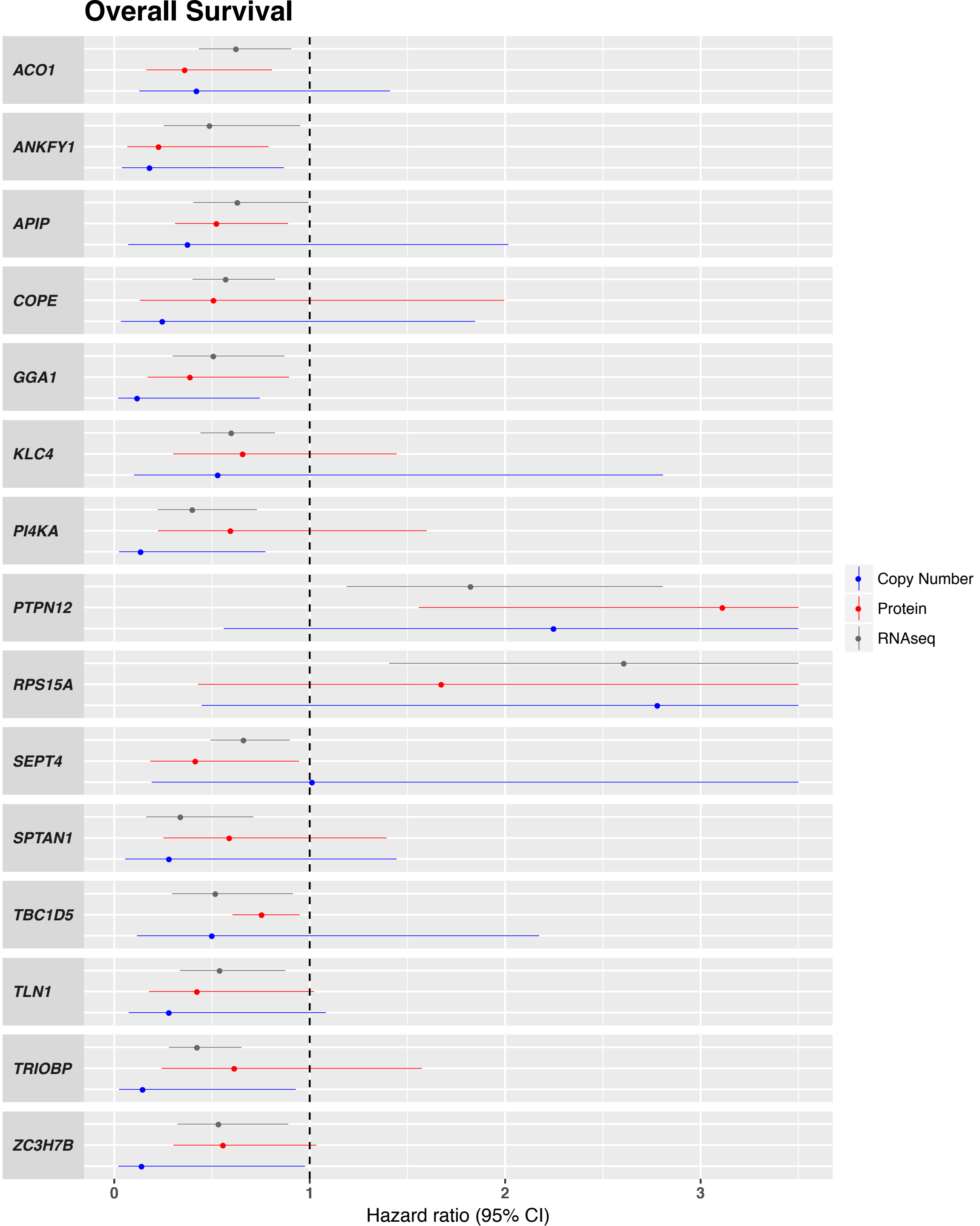

Supplementary Figure 8 – Meta-analysis of proteomic, RNAseq and CNV datasets. Forest plot showing 15 genes with impact on survival (Storey q-value <= 0.3). Hazard ratios (HR) above 1 describe increased risk with increased expression, while HR below 1 describe reduced risk with increased expression. The dots are point estimates of the HR and the lines on either side of the dot are 95% confidence interval. The upper limit of confidence intervals were truncated at 3.5.

Supplementary Figure 9

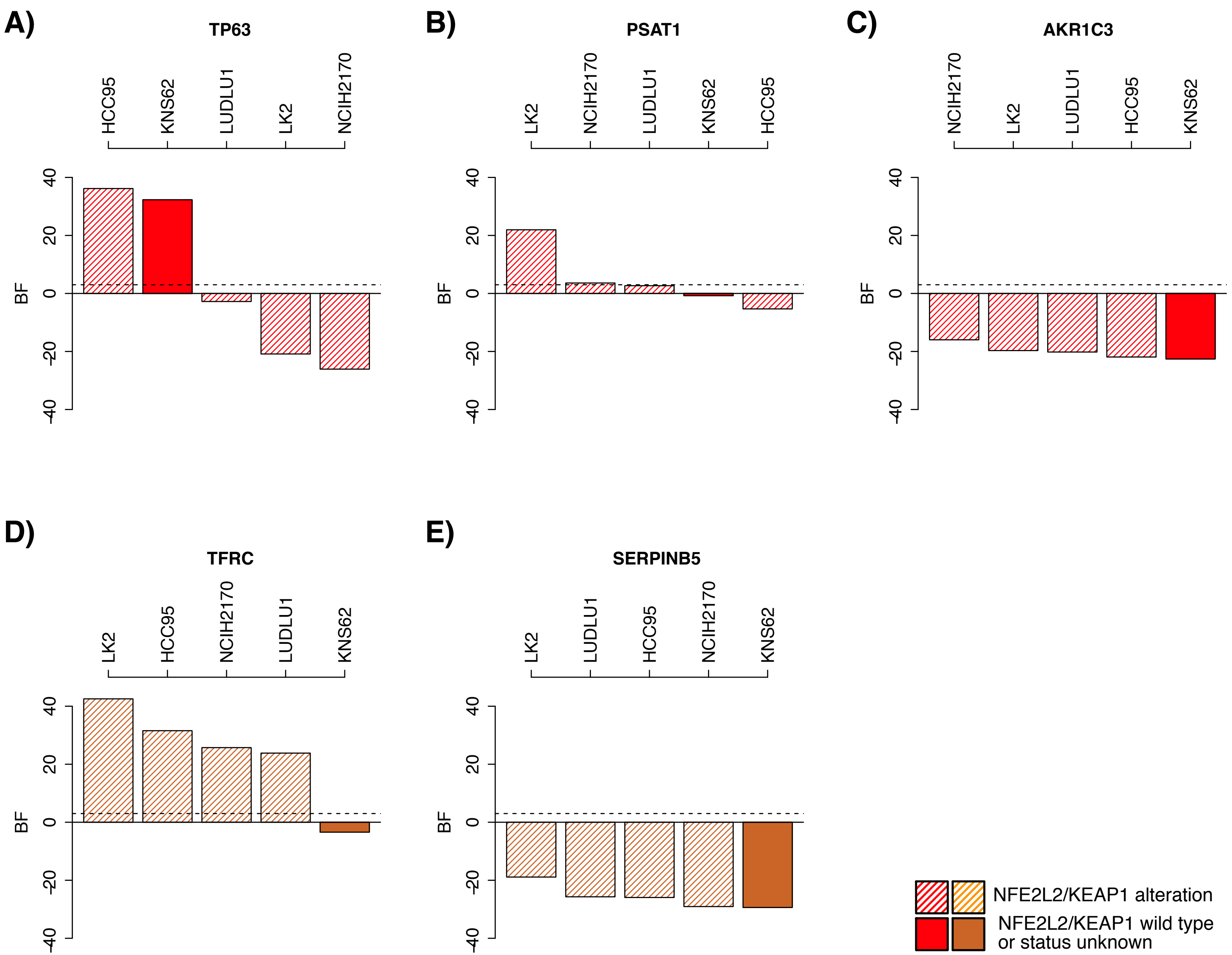

Supplementary Figure 9 – A-E) PICKLES results for the subset of target genes found in both Project DRIVE and PICKLES. A BF > 3 is considered significant.

Supplementary Figure 10

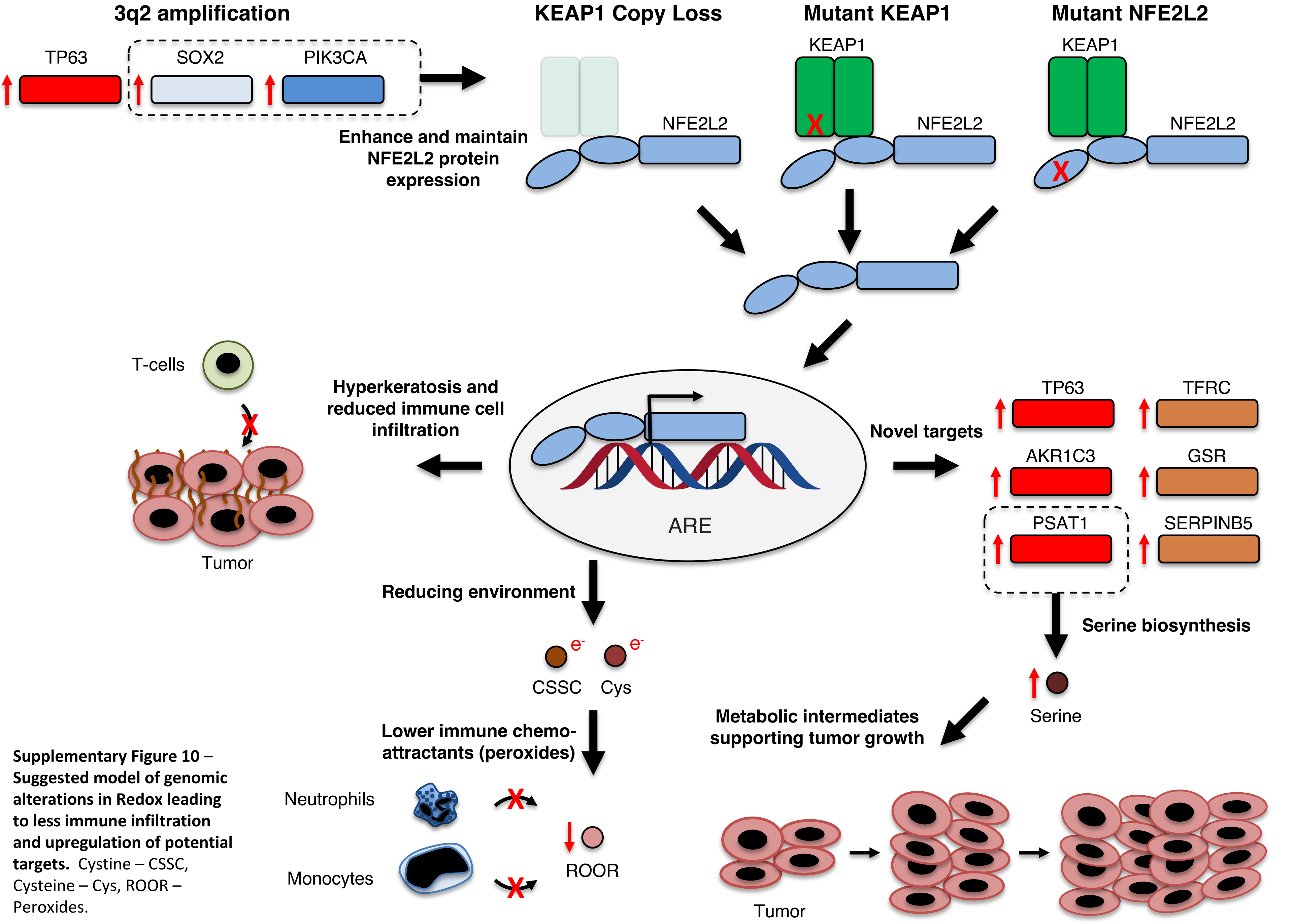

Supplementary Figure 10 – **Suggested model of genomic alterations in Redox leading to less immune infiltration and upregulation of potential targets.** Cystine – CSSC, Cysteine – Cys, ROOR – Peroxides.

Supplementary Figure 11

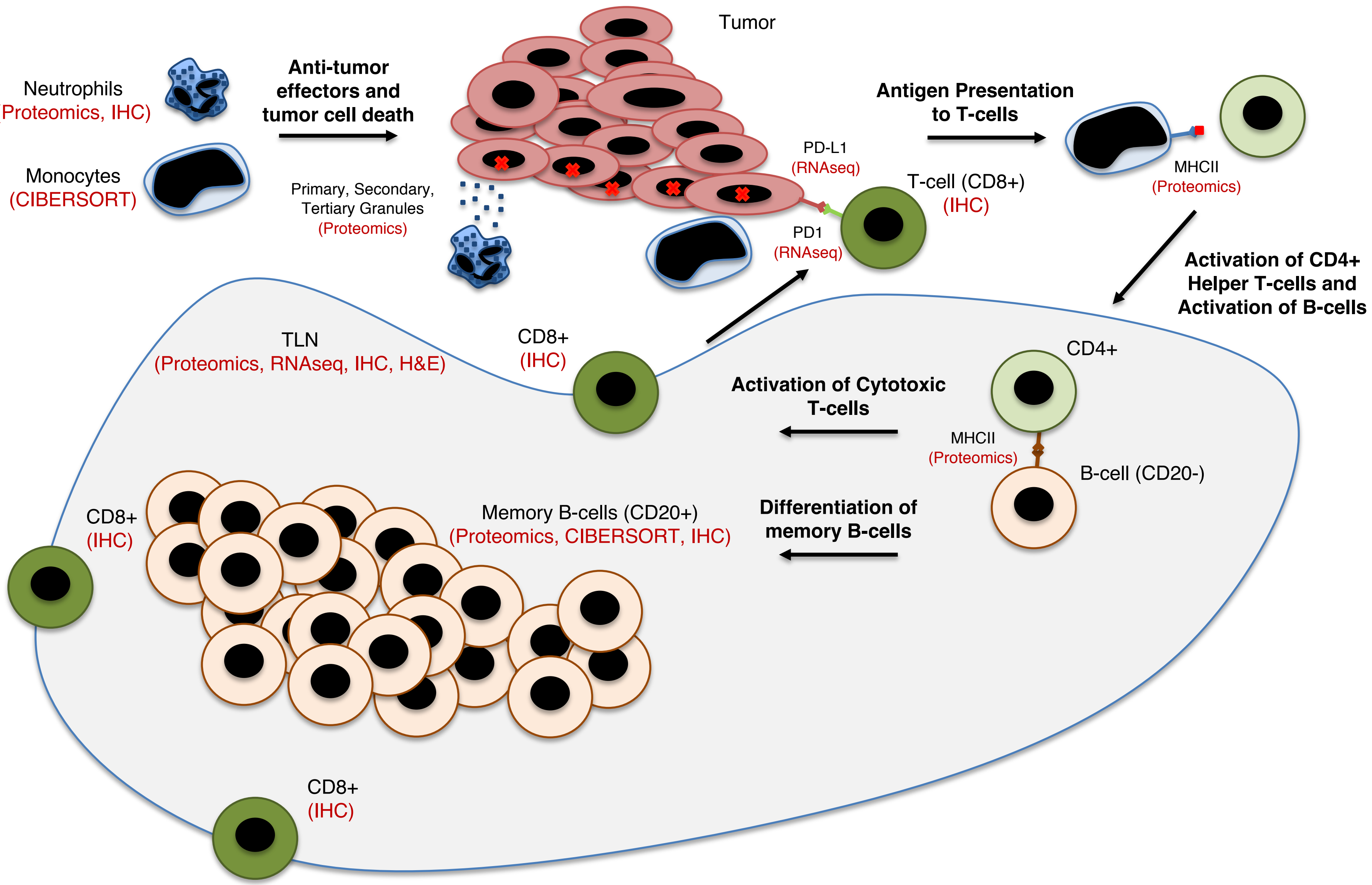

Supplementary Figure 11 – **Suggested model of inflammatory response in Inflamed tumors based on proteogenomic and immunohistochemical findings.**

Supplementary Figure 12

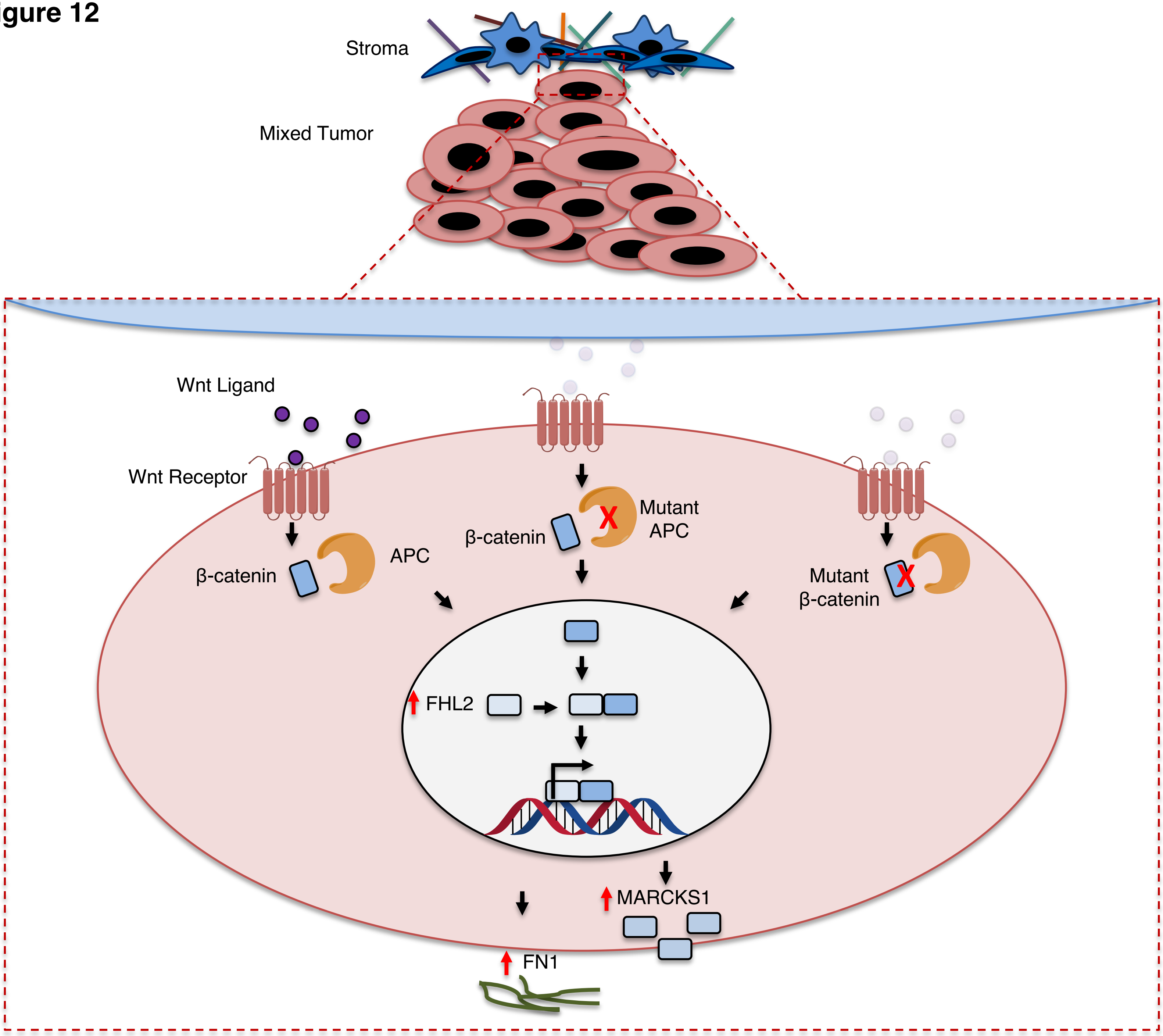

Supplementary Figure 12 – Suggested model of Wnt signaling in Mixed based on our experimental observations. There are several, subtle indications that Wnt signaling may play a role in Mixed.

Supplementary Figure 13

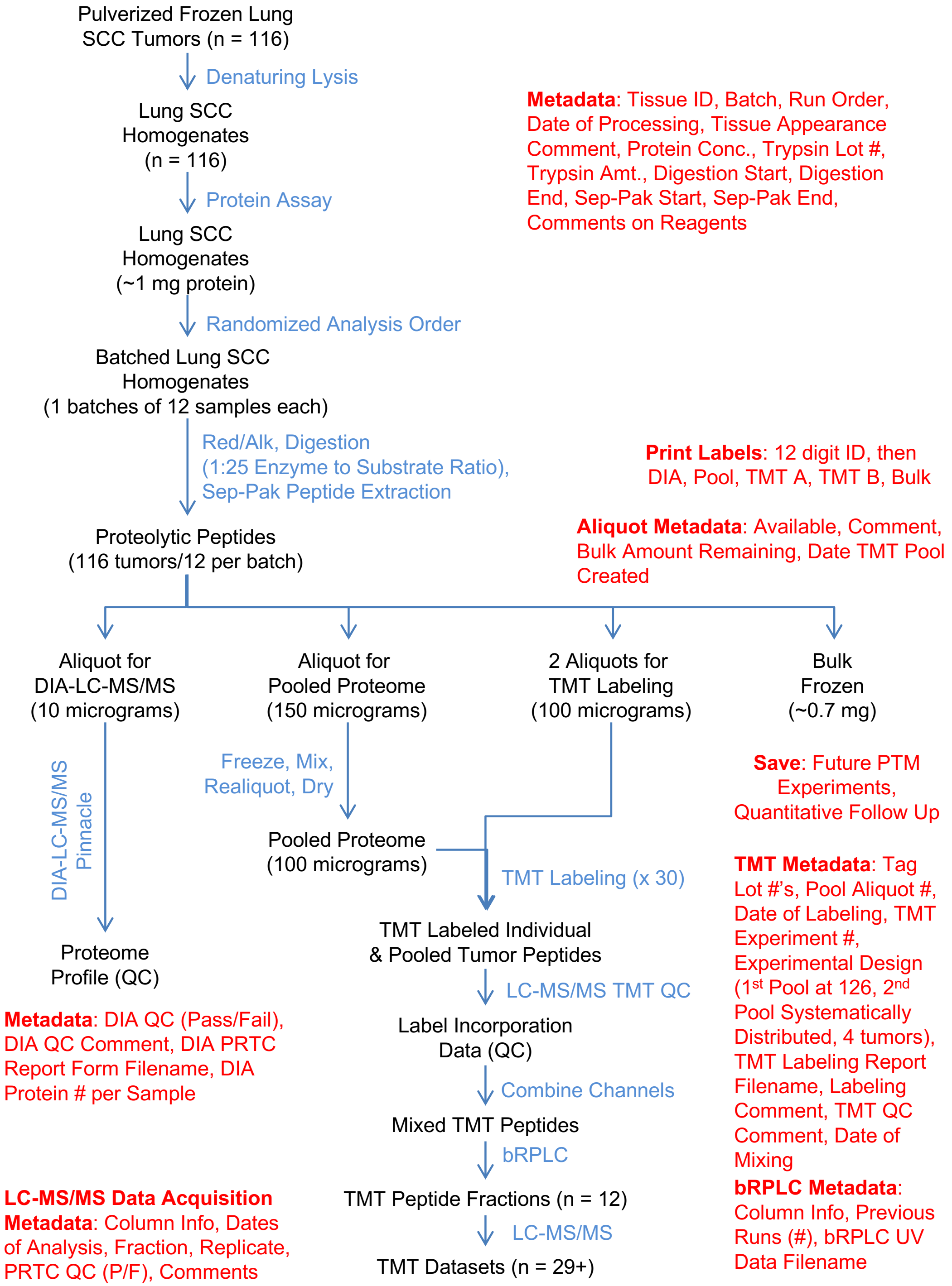

Supplementary Figure 13 – Workflow for Proteomics Analysis of Squamous Cell Lung Tumors. Outcomes are shown in black text and the method applied in each step is in blue text. Recorded metadata are listed in red for each experiment.

Supplementary Figure 14

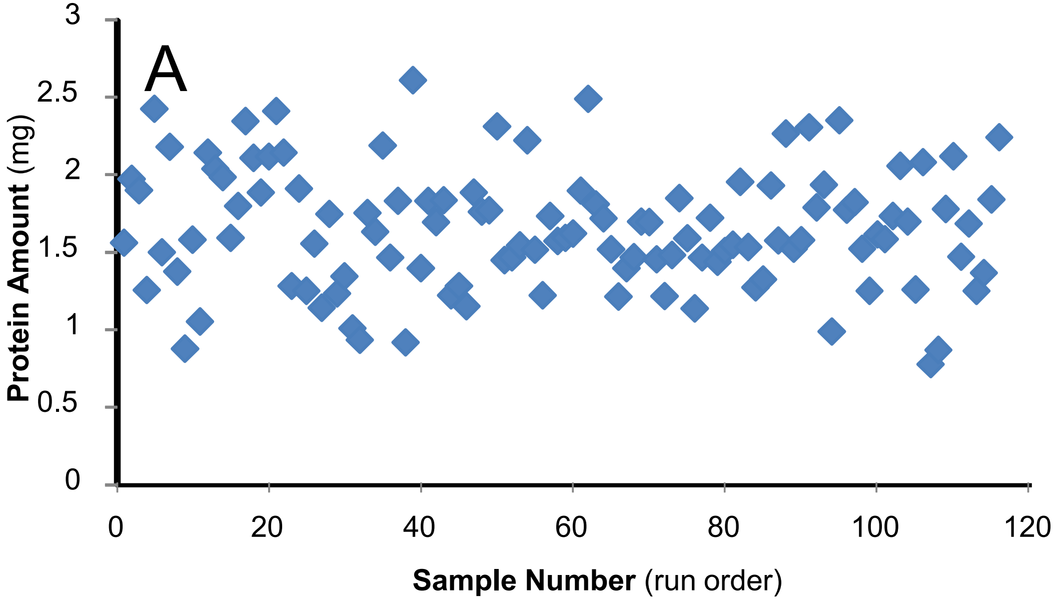

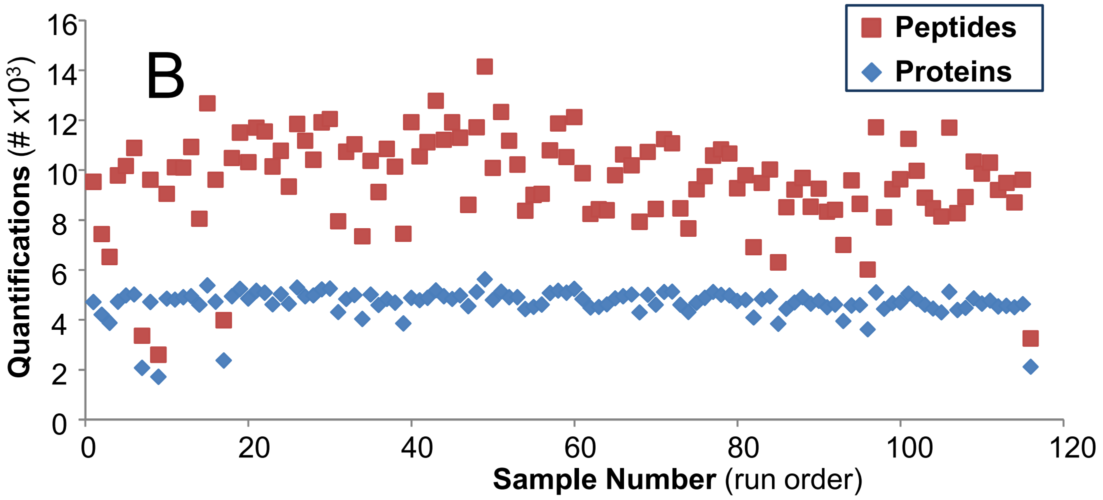

Supplementary Figure 14 – Extracted Protein Amount and Digestion Quality Control for Each Tumor Sample. A) The protein yield from each tumor homogenate and B) the number of identified peptides and proteins from LC-MS/MS analysis are shown. Four samples had lower amounts of total protein; re-analysis with LC-MS/MS ruled out poor instrument performance and indicated that these samples just have fewer observable proteins in this single sample analysis.

Supplementary Figure 15

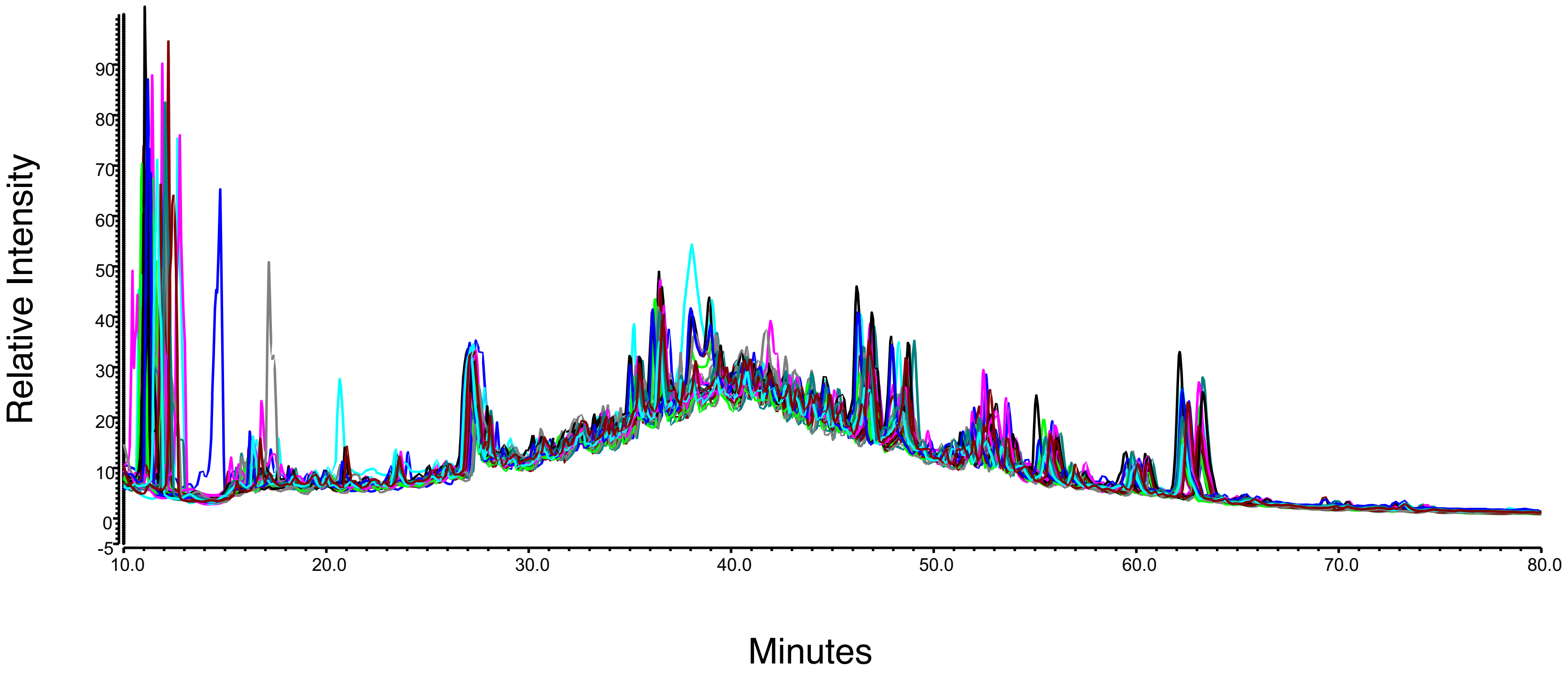

Supplementary Figure 15 – Reproducibility of Offline Peptide Fractionation. Chromatograms for each basic pH reversed phase separation are overlaid to illustrate the consistency of this step of the experiment.

Supplementary Figure 16

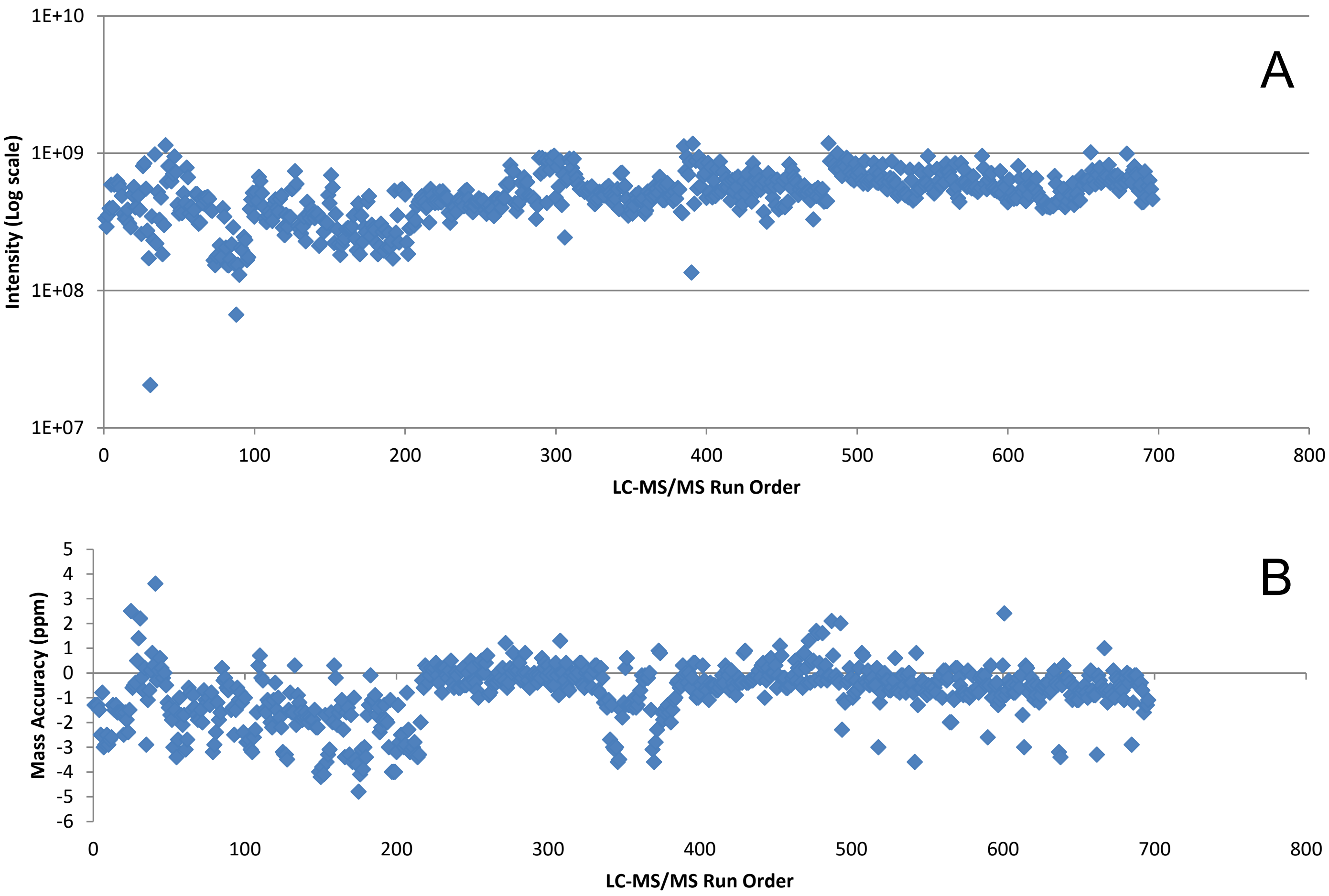

Supplementary Figure 16 – Reproducibility of Ion Signal and Mass Measurement Accuracy of Peptide Retention Time Calibrator Standards in Duplicate LC-MS/MS Analysis of 12 Peptide Fractions in 29 TMT 6-plex Experiments. A) Total PRTC peptide ion signal and B) average mass measurement accuracy in parts per million are plotted for each of 696 LC-MS/MS analyses.
