# Supplementary material for: Proteogenomic landscape of squamous cell lung cancer
Source: Nat Commun. 2019 Aug 8;10:3578. doi: 10.1038/s41467-019-11452-x (PMC6687710; doi:10.1038/s41467-019-11452-x)
Supplement: Supplementary file 3 — Description of Additional Supplementary Files [file 41467_2019_11452_MOESM3_ESM.pdf]

Description of Additional Supplementary Files

File Name: Supplementary Data 1
Description: Clinical and pathology.

File Name: Supplementary Data 2
Description: Targeted exome sequencing and copy number variation.

File Name: Supplementary Data 3
Description: Proteomics.

File Name: Supplementary Data 4
Description: RNAseq, ESTIMATE, and CIBERSORT.

File Name: Supplementary Data 5
Description: Association tests and tables.

File Name: Supplementary Data 6
Description: Correlation results.

File Name: Supplementary Data 7
Description: Proteomics differential expression.

File Name: Supplementary Data 8
Description: Enrichr GO pathway enrichment.

File Name: Supplementary Data 9
Description: MSigDB pathway enrichment.

File Name: Supplementary Data 10
Description: Project DRIVE results.
