# Supplementary material for: Proteogenomic landscape of squamous cell lung cancer
Source: Nat Commun. 2019 Aug 8;10:3578. doi: 10.1038/s41467-019-11452-x (PMC6687710; doi:10.1038/s41467-019-11452-x)
Supplement: Supplementary file 14 — Reporting Summary [file 41467_2019_11452_MOESM14_ESM.pdf]

nature research

Corresponding author(s): Eric Haura

Last updated by author(s): Jun 14, 2019

Reporting Summary

Nature Research wishes to improve the reproducibility of the work that we publish. This form provides structure for consistency and transparency in reporting. For further information on Nature Research policies, see Authors & Referees and the Editorial Policy Checklist.

Statistics

For all statistical analyses, confirm that the following items are present in the figure legend, table legend, main text, or Methods section.

n/a	Confirmed	
☐	☒	The exact sample size (*n*) for each experimental group/condition, given as a discrete number and unit of measurement
☐	☒	A statement on whether measurements were taken from distinct samples or whether the same sample was measured repeatedly
☐	☒	The statistical test(s) used AND whether they are one- or two-sided<br>*Only common tests should be described solely by name; describe more complex techniques in the Methods section.*
☐	☒	A description of all covariates tested
☐	☒	A description of any assumptions or corrections, such as tests of normality and adjustment for multiple comparisons
☐	☒	A full description of the statistical parameters including central tendency (e.g. means) or other basic estimates (e.g. regression coefficient) AND variation (e.g. standard deviation) or associated estimates of uncertainty (e.g. confidence intervals)
☐	☒	For null hypothesis testing, the test statistic (e.g. *F*, *t*, *r*) with confidence intervals, effect sizes, degrees of freedom and *P* value noted<br>*Give P values as exact values whenever suitable.*
☒	☐	For Bayesian analysis, information on the choice of priors and Markov chain Monte Carlo settings
☒	☐	For hierarchical and complex designs, identification of the appropriate level for tests and full reporting of outcomes
☒	☐	Estimates of effect sizes (e.g. Cohen's *d*, Pearson's *r*), indicating how they were calculated

Our web collection on statistics for biologists contains articles on many of the points above.

Software and code

Policy information about availability of computer code

Data collection	In-house data was used.
Data analysis	R version 3.4 was used for the integrative analyses detailed in the paper. Code is available here https://github.com/pstew/proteogenomics_scc . Methods contain description of the individual omics processing pipelines.

For manuscripts utilizing custom algorithms or software that are central to the research but not yet described in published literature, software must be made available to editors/reviewers. We strongly encourage code deposition in a community repository (e.g. GitHub). See the Nature Research guidelines for submitting code & software for further information.

Data

Policy information about availability of data

All manuscripts must include a data availability statement. This statement should provide the following information, where applicable:

- Accession codes, unique identifiers, or web links for publicly available datasets
- A list of figures that have associated raw data
- A description of any restrictions on data availability

We have provided extensive processed data as supplemental files to increase availability. Proteomic data have been uploaded to PRIDE (https://www.ebi.ac.uk/pride/archive/) with the accession PXD010357 and 10.6019/PXD010357 for the DIA QC data and accession PXD010429 and 10.6019/PXD010429 for the TMT data. Copy number data was uploaded to dbGaP (https://www.ncbi.nlm.nih.gov/gap/) and transcriptomic/genomic data was uploaded to SRA (https://www.ncbi.nlm.nih.gov/sra) under accession phs001781.v1.p1.

Field-specific reporting

Please select the one below that is the best fit for your research. If you are not sure, read the appropriate sections before making your selection.

☒ Life sciences ☐ Behavioural & social sciences ☐ Ecological, evolutionary & environmental sciences

For a reference copy of the document with all sections, see nature.com/documents/nr-reporting-summary-flat.pdf

Life sciences study design

All studies must disclose on these points even when the disclosure is negative.

Sample size	108 tumor tissue samples were used, one for each patient.
Data exclusions	No data was excluded.
Replication	One of the key findings from the paper, the existence of proteomic subtypes of squamous cell lung cancer, was identified by consensus clustering, which repeats clustering thousands of times. Findings were validated/strengthened by provided RNAseq and IHC results.
Randomization	Tissue samples were randomized by stage, recurrence, gender, vital status, and age prior to processing for omics experiments.
Blinding	Initial TLN pathology scoring via H&E was performed by a pathologist (TB) who was blinded to proteomic subtype.

Reporting for specific materials, systems and methods

We require information from authors about some types of materials, experimental systems and methods used in many studies. Here, indicate whether each material, system or method listed is relevant to your study. If you are not sure if a list item applies to your research, read the appropriate section before selecting a response.

Materials & experimental systems

n/a	Involved in the study
☐	☒ Antibodies
☒	☐ Eukaryotic cell lines
☒	☐ Palaeontology
☒	☐ Animals and other organisms
☒	☐ Human research participants
☒	☐ Clinical data

Methods

n/a	Involved in the study
☒	☐ ChIP-seq
☒	☐ Flow cytometry
☒	☐ MRI-based neuroimaging

Antibodies

Antibodies used	CD20 (Ventana 760-2531), CD8 (Ventana 790-4460), CD33 (Leica PA0555)
Validation	CD20 Immunohistochemistry assays (IHC) were run on the automated Ventana Discovery XT platform (Supplemental Table 1). Anti-CD20 (Ventana 760-2531), a mouse monoclonal (L26) was used at predilute concentration (0.3 [illegible]g/ml) with Cell Conditioning 1 solution for antigen retrieval along with a heated 16 minute primary incubation. OmniMap anti-Ms-HRP for 16 min was used for secondary incubation. Slides were counterstained with Ventana hematoxylin (760-2021) and Bluing Reagent (760-2037) for 4 min each. Ventana Ms IgG (760-2014) was used as the mouse IgG control and run under same conditions. Human tonsil tissue sections were used as both a positive and Ms IgG control.<br><br>CD8/CD33 was run as dual IHC stain on the automated Ventana Discovery XT platform (Supplemental Table 1). Anti-CD8 (Ventana 790-4460, predilute, 0.3 ug/ml) a rabbit monoclonal (SP57) was diluted 1:15 with Dako antibody diluent (S0809) to a final concentration of 0.02 ug/ml and anti-CD33 (Leica PA0555, predilute, 10 mg/ml), a mouse monoclonal (PWS44). The combined antibodies used Cell Conditioning 1 for antigen retrieval and a primary antibody heated incubation time of 40 minutes. Anti-CD8 was visualized using OmniMap DAB and anti CD33 was stained with Ultra Map-Red. OmniMap DAB secondary incubations were reacted for 4 min while UltraMap Red for 16 minutes. Ventana (Rb IgG) (760-1029) was used as the rabbit IgG control and Ventana (Ms IgG) (760-2014) was used as the mouse IgG control and run under same conditions. Human tonsil tissue sections were used as the positive control for CD8/CD33 staining.
